# Optical Identification of Parenteral Nutrition Solutions Exploiting Refractive Index Sensing

**DOI:** 10.3390/s22186815

**Published:** 2022-09-08

**Authors:** Valentina Bello, Elisabetta Bodo, Sabina Merlo

**Affiliations:** Department of Electrical, Computer and Biomedical Engineering, University of Pavia, Via Ferrata 5, 27100 Pavia, Italy

**Keywords:** artificial parenteral nutrition, healthcare device, refractive index sensor, nutrition solution identification, optofluidics, patient safety

## Abstract

Parenteral artificial nutrition (PAN) is a lifesaving treatment for a large population of patients affected by different diseases, and it consists of intravenous injection of nutritive fluids by means of infusion pumps. Wrong PAN solutions are, unfortunately, often administered, thus threatening the patients’ well-being. Here, we report an optofluidic label-free sensor that can distinguish PAN solutions on the basis of their volumetric refractive index (RI). In our system, a monochromatic light beam, generated by a laser diode, travels obliquely through a transparent, square-section polystyrene channel, is then back-reflected by a mirror, and finally exits the channel in a position that depends on the filling fluid RI. The displacement of the output light spot Δ*X_experim_* is easily detected with a linear, 1-D position sensitive detector (PSD). We initially calibrated the sensor with water-glucose solutions demonstrating a sensitivity *S* = Δ*X_experim_*/Δ*n* = 13,960 µm/RIU. We then clearly distinguished six commercial PAN solutions, commonly administered to patients. To the best of our knowledge, this is the first reported healthcare sensing platform for remote contactless recognition of PAN fluids, which could be inserted into infusion pumps to improve treatment safety, by checking the compliance to the prescription of the fluid actually delivered to the patient.

## 1. Introduction

Every human being needs nutrition and hydration to live. When a person cannot eat or drink or does not receive enough nutrients or fluids, then medical treatments are necessary to cover their vital need for nutrition. In this framework, the expression “artificial nutrition” (AN) is used to indicate therapeutic techniques and treatments applied to satisfy the nutritional demands of patients who are, temporarily or permanently, unable to feed themselves naturally and autonomously [1]. Even if it is not a medical topic very often discussed, artificial nutrition plays a fundamental role in the life of a large number of patients affected by different diseases. For example, artificial nutrition is usually administered to patients with inflammatory bowel disease to treat the deficiency of electrolytes and vitamins [2]. AN is also fundamental to oncology patients that suffer from malnutrition as a consequence of the cancer itself as well as of demolitive surgery and anti-tumor drugs [3]. Moreover, during the COVID-19 pandemic, for subjects intubated in the intensive care unit for more than 48 h, it has been shown that a correct AN with the supplementation of amino acids helps to lower the hyperinflammatory state and promotes normal physiological recovery [4,5]. Additionally, it must be stressed that AN is common among children and teenagers, too [6], and in particular, in the pediatric environment [7]. Two main methods to artificially deliver nutrition to patients exist. “Enteral artificial nutrition” refers to the delivery of substances into the gastrointestinal tract using a tube. In “parenteral artificial nutrition” (PAN), instead, liquid nutrients are infused intravenously by inserting a central catheter into the superior vena cava or into an arm vein. In both cases (enteral and parenteral nutrition), the nutrition mixtures are administered using automated programmable pumps for infusion.

PAN stays at the second place (after anti-infective drugs) with regards to medication errors in hospitals [8]: the scientific literature reports a great variety of human errors that can happen in parenteral artificial nutrition [9,10]. In more detail, as reported in [9], almost 75% of the errors occurs during transcription, ordering, labelling and administration of the therapy. For example, it often happens that the wrong PAN mixture is ordered through the hospital pharmacy computer system and then administered to the patient. In other cases, the parenteral nutrition label given to the patient deviates from the written order and sometimes the prescription by the caregiver is illegible, leading to misinterpretation or miscalculation [11,12,13]. Other common incidents include application of the wrong label onto a PAN mixture bag, incorrect preparation technique, omission of essential components or addition of incorrect ingredients. The consequences of errors in PAN can be very harmful to the patient’s health. Indeed, over the years, severe health issues and even deaths have been registered as a consequence of inconsistent parenteral nutrition practices related to errors in preparation, labeling, ordering, administration and monitoring [9]. For example, one fatal neonatal case was related to an excessive dose of zinc being added to PAN because of a compounding error [14]. Another work reported seven neonatal cases where PAN lacking in zinc have resulted in dermatitis, infection and one death [15]. Other relevant consequences are hyperglycemia, ions and micronutrient toxicity, heart problems, delay of discharge from hospital. Medications errors often occurs also in home PAN, where patients self-administer artificial nutrients [16,17]. In this framework, the consequences of incidents can be even more dangerous since qualified medical personal is not constantly at the patients’ side, constituting a big threaten for them. Hence, as many scientific papers stress, it is crucial the existence of standardized safety protocols [18,19] and of checking biomedical devices in order to improve the patient safety and reduce the rate of medications errors in the PAN process. Additionally, the presence of a control system could significantly improve the quality of life of patients treated with home PAN and their feeling of comfort toward the therapy.

**Figure 1:**
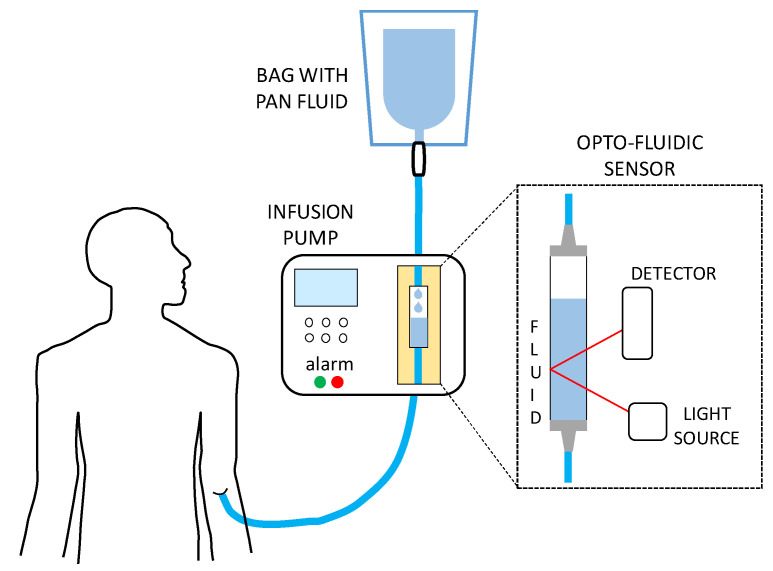
Schematic representation of the smart opto-fluidic sensor for recognition of PAN fluids and integration in commercial infusion pumps.

In this work, we report a smart optofluidic sensor that we have recently designed and developed, that is able to distinguish different types of commercial solutions used in parenteral artificial nutrition, on the basis of their volumetric refractive index (RI), bearing in mind that refractometric sensor are very popular and present many interesting features [20]. As described in the review article proposed by Singh [21], different methods for measuring the RI of various materials can be exploited. For example, fiberoptics sensors are used in a wide range of applications for RI detection [22,23]. However, since such sensors must be placed in contact with the fluid under test, they are not suitable for non-contact and non-invasive detection. Surface Plasmon Resonance (SPR) sensors allow to measure RI changes (also with a reference signal to compensate for temperature drifts) with high sensitivity but they usually require quite complex fabrication process and expensive components (such as moving optics or spectrophotometers) [24,25]. Recently, few authors proposed optical techniques based on the direction change of a refracted laser beam. However, many of these solutions still present some limitations, since they are not suitable for in-flow measurements and large volumes of fluids are often required. Moreover, expensive instrumentation is required. Our sensing platform was realized with low-cost optical and fluidic components, and it was designed to be easily integrated into commercial pumps for infusion, as shown in Figure 1. The operation of our sensor is based on the well-known principle of refraction of a light beam that propagates in different media [26,27,28,29,30]. In our system, light enters a rigid fluidic channel at a certain angle and a mirror is located behind it: then, the radiation is reflected and exits the channel in a different position with respect to the entrance point. In particular, if fluids with different refractive index fill the channel, the radiation is deflected at different angles inside the liquid (according to Snell law) and the light exit point shifts along the channel surface. This variation of position can be easily detected by means of a Position Sensitive Detector (PSD). After design and optimization of the electro-optofluidic configuration, we calibrated the sensor by testing water-glucose solutions (with the same range of RI of the considered PAN mixtures) and then we successfully exploited it to distinguish six commercial solutions that are used in actual situations for parenteral artificial nutrition. 

To the best of our knowledge, this is the first time a sensing platform is designed to distinguish PAN mixtures, suitable for integration into commercial automated infusion pumps, with the final aim of checking the compliance of the fluid being delivered to the patient’s body with the treatment prescribed by the caregiver.

## 2. Material and Methods

### 2.1. Solutions for Artificial Parenteral Nutrition

PAN solutions that were investigated and considered for experimental testing in this work contain amino acids, glucose, and electrolytes and have a water-like transparent appearance. Samples of transparent PAN mixtures were provided by the pharmacy of the I.R.C.C.S. San Matteo (Pavia, Italy) and belong to the commercial families CLINIMIX (Baxter S.r.l., Deerfield, IL, USA) and AMINOMIX (Fresenius Kabi, Bad Homburg, Germany). The amino acids with the electrolytes and the glucose are initially contained in two different compartments divided by a plastic membrane. The membrane can be easily broken to mix the liquids just before administering the PAN fluid to the patient. Every type of CLINIMIX and AMINOMIX contains amino acids and glucose in variable concentrations, resulting in different amounts of calories. Hence, it is reasonable to suppose that every mixture has a different refractive index, that can be considered as the characteristics parameter to distinguish the liquids. Thus, the theoretical RI value of every PAN fluid (*n_PAN fluid_*) was estimated by computing the formula

(1) $$n_{PAN\;fluid}=n_{H2O}+k_{glu}\cdot C_{glu}+\;\overset{Na}{\underset{i=1}{\sum }}k_{amino}\left(i\right)\cdot C_{amino}\left(i\right)+\overset{Ne}{\underset{j=1}{\sum }}k_{electro}\left(j\right)\cdot C_{electro}\left(j\right)$$

where *n_H_*_2*O*_ is the RI of water, *k_glu_* = 0.00143 × 100 mL/g is the RI increment coefficient for glucose [31], *C_glu_* is the concentration of glucose, *N_a_* is the total number of amino acids, *k_amino_*(*i*) is the RI increment coefficient due to the *i*-th amino acid expressed in mL/g, *C_amino_*(*i*) is the concentration of the *i*-th amino acid expressed in g/mL, *N_e_* is the total number of electrolytes, *k_electro_*(*j*) is the RI increment coefficient due to the *j*-th electrolyte expressed in mL/g, *C_electro_*(*j*) is the concentration of the *j*-th amino acid expressed in g/mL. Values of the RI increment coefficient for every amino acid and electrolyte are tabulated in the literature in [32,33], respectively. Moreover, the exact concentration of each substance in every PAN fluid is reported on the leaflet of each bag. Hence, the RI of every available AN fluid could be theoretically estimated at a specific wavelength, such as 670 nm that is the emission wavelength of the laser source used for the optical readout (see Section 4). Table 1 clearly shows that every PAN mixture is characterized by a different RI value: therefore, the refractive index is a suitable parameter to be monitored to distinguish PAN fluids.

### 2.2. Optofluidic Configuration

**Figure 2:**
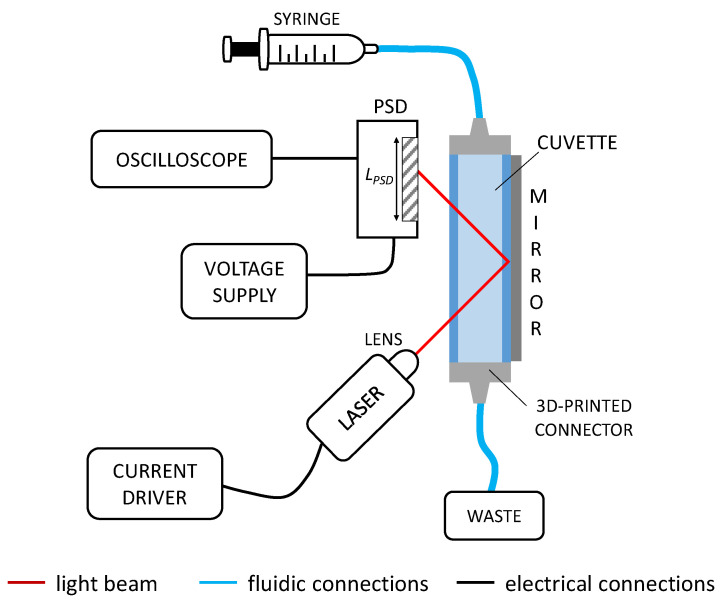
Optofluidic instrumental configuration of the sensing platform. PSD: Position Sensitive Detector.

The instrumental configuration of the optofluidic sensing platform is reported in Figure 2. The sample contained in a syringe is pushed inside a plastic cuvette used as fluidic channel. It is a standard 1 cm × 1 cm polystyrene cuvette traditionally used in spectrophotometers (Cheimika S.A.S, Pellezzano, Italy). It has length *L* = 5 cm, channel width *d* = 10 mm, wall thickness *t* = 1 mm (nominal values) with RI of 1.5862 RIU. The cuvette was customized by polishing the bottom side and adding two 3D-printed plastic connectors for interconnection with plastic tubes. All measurements were performed in static conditions. The readout radiation is provided by a semiconductor laser diode (HL6748MG, Thorlabs, Newton, NJ, USA) with emission wavelength at 670 nm and maximum optical power of 10 mW, powered by a bench current driver (LDC 500, Thorlabs, Newton, NJ, USA). A glass lens (LTN330-B, Thorlabs, Newton, NJ, USA) with focal length of 3.1 mm, numerical aperture of 0.68 and anti-reflection coating is placed in front of the source and the light beam is focused in order to obtain the smallest light spot onto the surface of the detector. Light beam is shined onto the flat surface of the fluidic channel with an angle of incidence of 45°. The radiation crosses twice the cuvette containing the sample under test, bouncing once on a commercial low-cost bulk mirror (12.5 mm × 50 mm, thickness of 1 mm), (RUINUO, Shijiazhuang, China, available from Amazon, Seattle, WA, USA) and glued to the cuvette back wall, by adding some glue along the mirror borders. After crossing the fluid, the light beam reaches a one-dimensional linear Si-PSD (1L10_CP2, by SiTek Electro Optics AB, Partille, Sweden) that is oriented parallel to the cuvette, on the same side of the laser, at a distance of few millimeters. The Si-PSD active surface is 10 mm (*L_PSD_*) × 2 mm and the overall chip has an area of 19.6 mm × 7.4 mm. The detector is powered with a voltage supply (PS283, Tektronix, Beaverton, OR, USA) between −15 V and +15 V. The PSD provides two photogenerated currents (*I*_1_ and *I*_2_) as output signals; hence, a transimpedance circuit was designed and realized to convert the current signals in voltage signals (*V*_1_ and *V*_2_) and amplify them. In particular, *V*_1_ and *V*_2_ are proportional to *I*_1_ and *I*_2_ and depend on the position of the light spot: if it is located at the center of the sensing region, then *V*_1_ = *V*_2_. The voltage output signals supplied by the PSD are visualized in real-time and then acquired with an oscilloscope (MDO3034, Tektronix, Beaverton, OR, USA) with USB connection. Eventually, data are processed in MATLAB environment.

### 2.3. Principle of Operation of the Sensor

**Figure 3:**
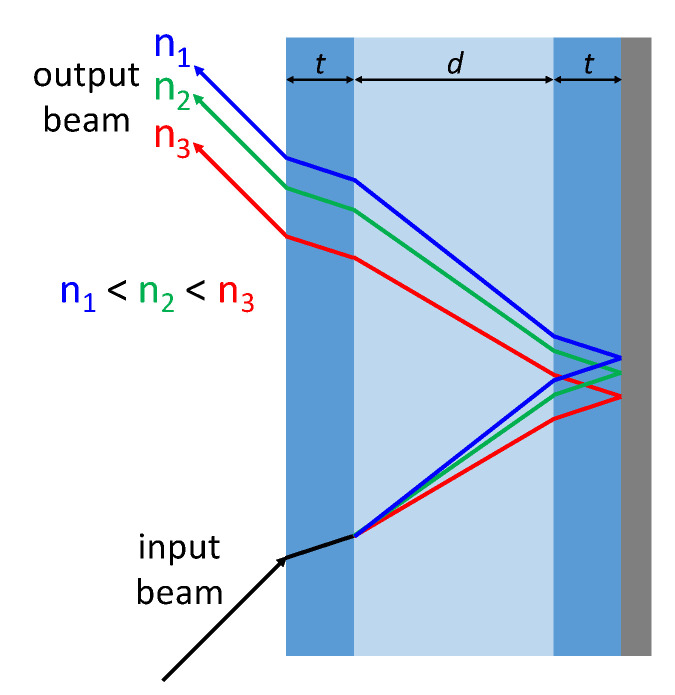
Schematic representation of the sensor operation when different samples with increasing RI are considered in the channel.

As already anticipated, the principle of operation of the sensor is simple: it is based on the observation that, if a laser beam enters a rigid fluidic channel at a certain angle and a mirror is located behind it, then the radiation is reflected and exits the channel at a different position with respect to the entrance point (Figure 3). The exit position depends on the refractive index of the liquid filling the channel: indeed, the radiation is deflected at different angles inside the liquid (according to Snell law), and the light exit point shifts along the channel surface. In particular, if the RI of the fluid increases, the output beam shifts along the channel surface and becomes closer to the input radiation. This variation of position is then detected by the PSD.

## 3. Results

**Figure 4:**
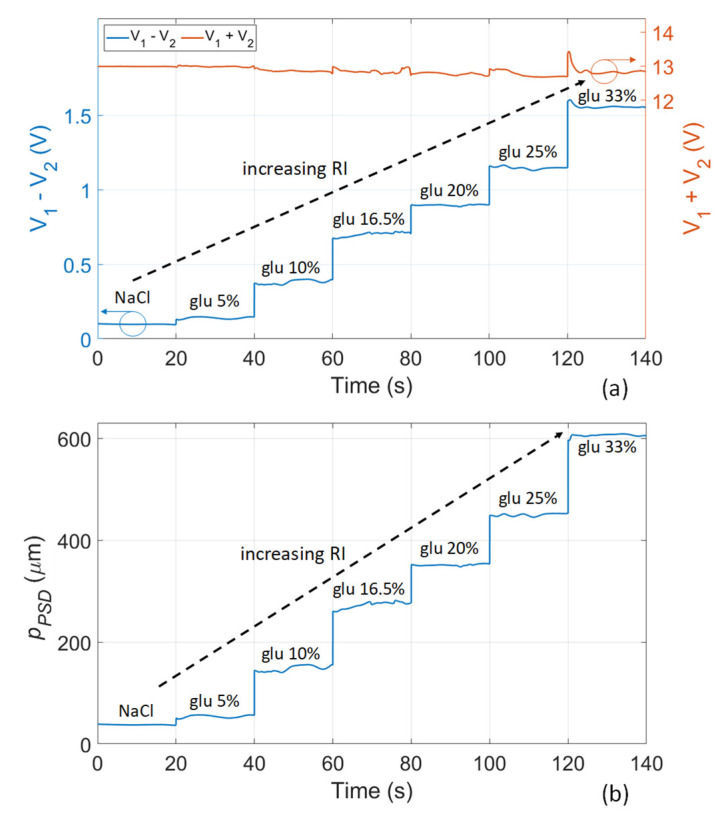
Experimental signals in the time domain obtained during calibration of the optofluidic platform with NaCl and glucose–water samples: (**a**) difference *V*_1_ − *V*_2_ (blue trace) and sum *V*_1_ + *V*_2_ (orange trace); (**b**) position of the laser beam *p_PSD_* measured by the PSD and retrieved by computing Equation (2).

In order to prove the suitability of the proposed readout method and of the optofluidic platform for detecting different fluids on the basis of their refractive index and to obtain a full calibration of the sensor, preliminary experimental measurements were performed by flowing a solution of 0.9% sodium chloride (NaCl) and dilutions of glucose in deionized water in different concentration inside the cuvette. The physiological solution of NaCl was used to simulate a realistic situation where the sensing platform is always initially calibrated using a reference fluid that is intravenously injectable. Seven samples with increasing RI in the interval 1.3325–1.3788 RIU were employed to calibrate the platform in the same range of RI of the considered NAD solutions (Figure 3). The laser current was set equal to 23.7 mA. All measurements were carried out at a constant room temperature of about 25 °C with fluctuations of ± 0.5 °C, which do not affect the fluid RI in a significantly way. When different fluids fill the cuvette, the position of the output beam changes; thus, using the PSD it is possible to measure this variation of position, as previously explained. In particular, it is significant to consider the difference signal *V*_1_ − *V*_2_ and the sum signal *V*_1_ + *V*_2_ (blue and orange trace, respectively, in Figure 4a). The difference signal carries information about the beam position and, thus, its value changes for different sample RIs. The sum signal is proportional to the total optical power reaching the PSD sensitive surface; although it remains in general constant, it is required to normalize the difference signal and compensate for spurious changes of light intensity on the detector. The light spot position on the PSD (*p_PSD_*), with respect to the center of the sensitive area, can be retrieved by computing the formula

(2) $$p_{PSD}=\frac{L_{PSD}}{2}\times \frac{\left|V_{1}\right|-\left|V_{2}\right|}{\left|V_{1}\right|+\left|V_{2}\right|}$$

Figure 4b shows the displacement of the beam for increasing RI value of the samples.

Afterwards, the experimental shift *X_experim_* of the light beam position with respect to that measured when NaCl (chosen as reference fluid) is in the cuvette was retrieved, for each sample investigated, by applying the formula

(3) $$X_{experim}=\left|p_{PSD\;fluid}-p_{PSD\;NaCl}\right|$$

**Figure 5:**
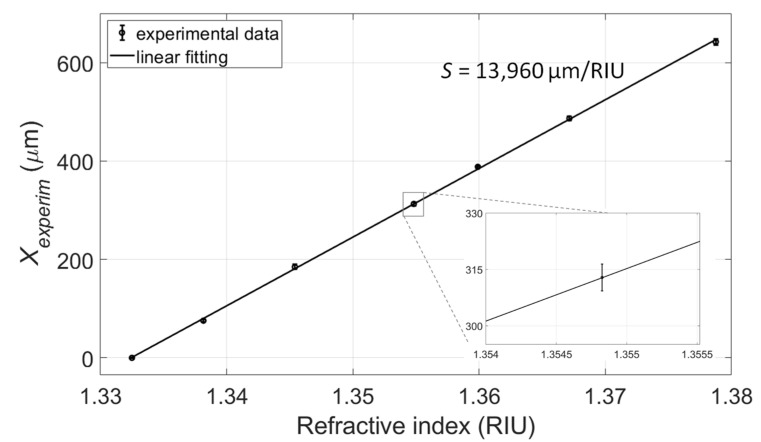
Linear calibration curve of the sensor obtained by fitting the values of *X_experim_* as a function of the RI.

The values of *X_experim_* as functions of the RI were then linearly fitted and the calibration curve of the platform was obtained (Figure 5), yielding a sensitivity (defined as *S* = Δ*X_experim_*/Δ*n*, where *n* is the RI) of 13,960 µm/RIU (determination coefficient of the fitting *R^2^* = 0.998). The calibration curve has equation

(4) $$X_{experim}\left[µm\right]=13960\;\left[µm/\mathrm{RIU}\right]\times n\;\left[\mathrm{RIU}\right]-18600\;\;\left[µm\right]$$

**Figure 6:**
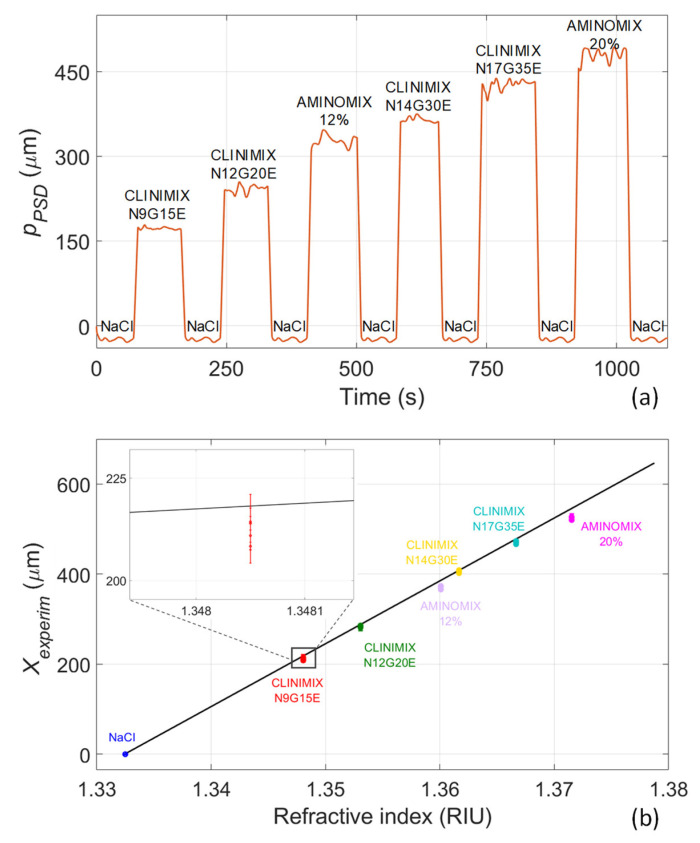
Experimental testing of PAN mixtures. (**a**) Beam position *p_PSD_* for reference solution (NaCl) and PAN fluids. (**b**) *X_experim_* for the tested PAN fluids in comparison with the calibration curve. In the inset, a zoomed view is reported, showing the four repetitions of the measurement.

After the calibration procedure, PAN fluids were tested. Samples were prepared by mixing amino acids and electrolytes with glucose (initially contained in different compartments of the bag) just a few minutes before starting the experiments. Measurements were repeated in three different days within a week since the preparation of the samples. After opening, bags with PAN mixtures were stored in a refrigerator at 4 °C. To simulate a realistic situation where the sensing platform is always initially calibrated using a reference fluid that is intravenously injectable, measurements were carried out by first filling the cuvette with NaCl and immediately after with the PAN fluid. The procedure was repeated for all the six available PAN mixtures. The position of the light beam *p_PSD_* measured by the PSD for all the samples tested is reported in Figure 6a. The beam position obtained when testing NaCl solution is repeatable, while it varies when testing PAN fluids with increasing RI. Then, the experimental shift of the light beam position *X_experim_* was retrieved for every PAN fluid; it is reported as a function of the theoretical RI (see Table 1) in Figure 6b and its behavior is compared with the calibration curve previously obtained (see Figure 5). The experimental data are well distributed along the calibration curve that can be correctly used to detect the RI of the PAN fluids. Testing of the PAN mixtures was repeated 4 times, as can be better appreciated in the zoomed inset of Figure 6b: the repeatability of the measurement is good.

## 4. Discussion

**Figure 7:**
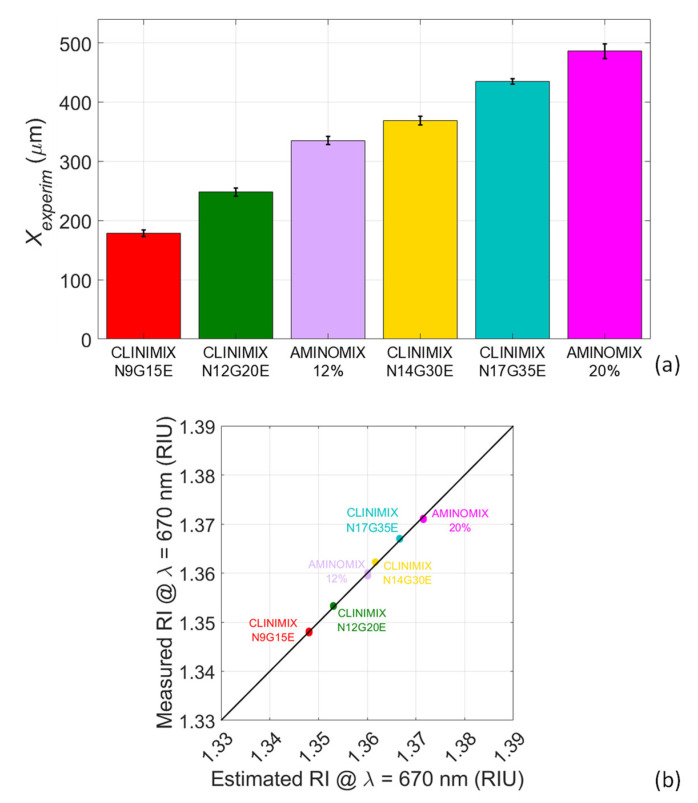
Results of experimental testing of PAN mixtures. (**a**) Bar chart representing the experimental shift *X_experim_* for every PAN fluid. (**b**) Comparison between the RIs measured by the sensor and the estimated RIs.

The experimental shift obtained for the six PAN fluids is reported in the bar chart of Figure 7a to compare the sensor response obtained after the testing of each solution. The height of each bar represents the average value of *X_experim_*, while the black segments have a length that is equal to twice the standard deviation. The bar chart highlights that every PAN mixture can be distinguished from all the others since the values of the experimental shift are significantly different, also considering their standard deviations. These results are really promising, and they suggest the suitability of the proposed technique to distinguish PAN solutions in a remote contactless manner. Eventually, the RI of the PAN mixtures measured with the opto-fluidic sensor was retrieved by inverting the equation of the calibration curve (Equation (4)). These values were then compared with the theoretical values estimated at 670 nm (reported in Table 1), as reported in Figure 7b. Each couple of values measured RI-estimated RI is well superimposed to the black line that represent the bisector, meaning that the measured values of RI are in a very good agreement with the predictions obtained by computing Equation (1). These data also prove that the accuracy of the sensing platform is very good: indeed, the absolute error, calculated as absolute value of the difference between the measured RI values and the theoretical values expected, is in the range between 6 × 10^−4^ and 4 × 10^−5^ RIU. Hence, it is very small with respect to the minimum difference between the refractive indices of the considered solutions.

## 5. Conclusions

PAN plays a fundamental role in the life of many patients and, unfortunately, medication errors related to this type of therapy are still very common. In this work, for the first time to our knowledge, we have reported an optofluidic label-free sensor based on refractometric measurements to distinguish transparent nutrient mixtures employed in PAN and containing water, glucose, amino acids, and electrolytes. The different composition of the PAN fluids allows to identify them on the basis of their RI. The sensor includes a diode laser (~€25) and a rigid, disposable fluidic channel. A disposable, plastic cuvette (~€0.10) and a cheap mirror (~€0.20) were used in view of developing a low-cost device. The position of the output beam depends on the liquid filling the channel and it is detected by means of a PSD (~€50). The optofluidic platform was first calibrated using water–glucose solutions (proving a sensitivity of 13,960 µm/RIU) and it was successfully exploited to recognize with high selectivity six commercial PAN mixtures. The sensor can be easily inserted along the fluidic path connecting the bag containing the fluid to the patients’ blood vessel. Moreover, we are currently designing a 3D printed mounting for an easier integration of all optical and fluidic components to realize an ultra-compact optofluidic platform to be incorporated in automated pumps for infusion. The bulk current driver will be substituted by a microcontroller mounted on a small electronic board to make the experimental setup much more compact. The final prototype could also include a temperature sensor in order to compensate for small RI drifts due to thermal effects. Since another severe complication of PAN is embolism, as a future perspective, we are investigating the use of the same platform for real-time simultaneous detection of air bubbles that can generate along the fluidic path with severe consequences for the patients’ security. Eventually, future work will be also devoted to develop a suitable readout technique for the analysis of PAN fluids also containing lipid particles. Indeed, these mixtures are highly diffusive, and they are not suitable to be analyzed with the proposed optical technique.

## Figures and Tables

**Table 1:** List of commercial PAN solutions provided by the pharmacy of the I.R.C.C.S. San Matteo for experimental testing. Commercial names (first column); theoretical RI values estimated by Equation (1) at the wavelength of 670 nm (second column).

PAN Fluid	*n_PAN fluid_* (RIU) Estimated at 670 nm
CLINIMIX N9G15E	1.3481
CLINIMIX N12G20E	1.3531
AMINOMIX 20%	1.3601
CLINIMIX N14G30E	1.3617
CLINIMIX N17G35E	1.3667
AMINOMIX 20%	1.3716

## Data Availability

The data presented in this study are available on request from the corresponding author.
